# Green and sustainable synthesis of CaO nanoparticles: Its solicitation as a sensor material and electrochemical detection of urea

**DOI:** 10.1038/s41598-023-46728-2

**Published:** 2023-11-15

**Authors:** T. S. Sunil Kumar Naik, Simranjeet Singh, Pavithra Narasimhappa, Radhika Varshney, Joginder Singh, Nadeem A Khan, Sasan Zahmatkesh, Praveen C. Ramamurthy, Nabila Shehata, G. N. Kiran, K. Sunil

**Affiliations:** 1 Department of Materials Engineering, Indian Institute of Science, Bangalore, 560012 India; 2 Interdisciplinary Centre for Water Research (ICWaR), Indian Institute of Science, Bangalore, 560012 India; 3 Department of Botany, Nagaland University, Lumami, Nagaland 798627 India; 4 Interdisciplinary Research Center for Membranes and Water Security (IRC-MWS), King Fahd University of Petroleum and Minerals, Dhahran, 31261 Saudi Arabia; 5 Tecnologico de Monterrey, Escuela de Ingenieríay Ciencias, Puebla, Mexico; 6 Environmental Science and Industrial Development Department, Faculty of Postgraduate Studies for Advanced Sciences, Beni-Suef University, Beni-Suef, Egypt; 7 Department of Chemistry, SSIT, Sri Siddhartha Academy of Higher Education, Tumkur, Karnataka 572107 India

**Keywords:** Environmental chemistry, Environmental impact

## Abstract

Urea is recognized as one of the most frequently used adulterants in milk to enhance artificial protein content, and whiteness. Drinking milk having high urea concentrations which causes innumerable health disputes like ulcers, indigestion, and kidney-related problems. Therefore, herein, a simple and rapid electroanalytical platform was developed to detect the presence of urea in milk using a modified electrode sensor. Calcium oxide nanoparticles (CaO NPs) were green synthesized and used as a catalyst material for developing the sensor. Synthesized materials formation was confirmed by different techniques like FTIR, UV–visible, XRD, SEM–EDX, and Raman spectroscopy. The carbon paste electrode (CPE) was modified using the CaO NPs and used as a working electrode during the analysis followed by cyclic voltammetry and differential pulse voltammetry (DPV) techniques. The fabricated calcium oxide modified carbon paste electrode (CaO/CPE) successfully detected the presence of urea in the lower concentration range (lower limit of detection (LLOD) = 0.032 µM) having a wide linear detection range of 10–150 µM. Adsorption-controlled electrode process was achieved at the scan rate variation parameter. The leading parameters like the selectivity, repeatability, and stability of the CaO/CPE were investigated. The relative standard deviation of sensor was ± 3.8% during the interference and stability study.

## Introduction

A non-protein nitrogen source carbonyl diamide (commonly known as urea), is one of the most frequently detected adulterants present in milk and dairy products. Introducing urea into adulterated milk increases its artificial protein content and enhances whiteness and consistency^1–3^. Being water-soluble and an end product of nitrogen metabolism in the body, urea is naturally present in milk in the range of 180–400 ppm^1,4,5^. As per the Food Safety and Standard Authority of India (FSSAI), the maximum permissible concentration of urea in milk is 700 ppm^6^. Detection of urea in milk above 700 ppm is often an indicator of the detrimental health of the milk-producing animal or milk adulteration. Intake of milk having high urea concentrations can result in various health issues such as ulcers, indigestion, acidity, intestinal damage, and kidney-related issues^3,5,7^. Urea has also been linked to anemia, insulin resistance, and the stimulation of oxidative stress^8^. Therefore, developing a sensitive and selective methodology for detecting urea is essential.

Over the years, various techniques have been employed to detect the amount of urea present in milk, such as Raman spectroscopy^9^, Fluorescence spectroscopy^10^, Liquid chromatography-mass spectrometry (LC–MS)^11^, Fourier transform-infrared spectroscopy (FTIR)^12^, and Surface plasmon resonance (SPR)^13^. However, these techniques are capital-intensive, demanding sophisticated instruments and trained personnel to operate. Further, they often require milk samples to be pre-treated before the detection of urea can be carried out. On the other hand, the electrochemical sensing platform offers a low-cost solution to effectively detect urea from milk samples with simplicity, selectivity, and high sensitivity, within seconds^7,14^. Electrochemical sensing is based on the measurement of the current generated at an applied potential in an electrochemical system owing to the redox reactions occurring at the working electrode interface. A series of working electrodes such as carbon paste electrodes (CPE), glassy carbon electrodes (GCE), and graphite electrodes (GE) have been used and are often modified with various materials to enhance their sensitivity and selectivity toward the target analyte. Urea can be detected using both enzymatic as well as non-enzymatic approaches. The enzymatic approach involves the immobilization of enzymes, mainly urease, onto the working electrode that further detects urea by converting urea into ammonium ions. However, involving enzymes, this approach is costly, requires optimum operating conditions, lacks stability, requires low storage temperatures for electrodes, and has limited reusability. On the other hand, non-enzymatic approaches are more stable and robust to be employed for the detection of urea^14–16^.

Non-enzymatic approaches often involve modification of working electrodes using nanoparticles such as carbon nanotubes, metal–organic frameworks, graphene oxide, and metal oxide nanostructures^15–19^. Among them, nanostructures based on metal oxide are one the vital candidates for modification of working electrodes owing to their large surface area, non-toxicity, high chemical stability, effective electron conductivity, and fast response time^20^. According to the literature review, CaO nanoparticles (CaO NPs) have not been reported for the electrochemical sensing of urea. Calcium oxide nanoparticles can be synthesized using natural precursors such as eggshells^21^, shrimp shells^22^, oyster shells^23^, and plant extracts^24–26^. Recently our group succeeded in immobilizing biogenic CaO NPs synthesized from seashells on the surface of graphene oxide, followed by its use in effectively detecting and removing Cr (VI) from water^27^. Therefore, CaO NPs can be employed for electrochemical detection of other target analytes such as urea.

In the present work, CaO NPs have been synthesized utilizing a green precursor, Lala clam seashells, by sol–gel method followed by calcination. Seashell-extracted CaO NPs were then characterized by various techniques such as scanning electron microscopy (SEM), X-ray diffraction (XRD), and Fourier transform-infrared spectroscopy (FTIR). Further, CPE was modified with sea shell extracted CaO NPs for the electrochemical sensing of urea using CV and DPV techniques. The developed sensor for urea demonstrated remarkable sensitivity, with excellent reproducibility, and was tested on real-world samples as well. In addition, the conceivable interaction sites between the urea and CaO NPs were prophesied using the Discovery Studio visualizer.

## Experimental section

### Chemicals

Urea (CH_4_N_2_O), potassium hydroxide (KOH), fine graphite powder, silicone oil, glucose (C_6_H_12_O_6_), ascorbic acid (C_6_H_8_O_6_), citric acid (C_6_H_8_O_7_), glycine (C_2_H_5_NO_2_), oxalic acid (C_2_H_2_O_4_), and thiourea (CH_4_N_2_S) were procured from Sigma-Aldrich (Bangalore, India). Clamshell was accumulated from Panamburu beach, located in Mangalore district (Karnataka India). Potassium hydroxide solution was used as a supporting electrolyte. All the stock solutions were prepared using ultra-Millipore water.

### Equipment

A model CHI-660c potentiostat (CH Instrument-660 electrochemical workstation) with a typical three-electrode cell was used to conduct all electrochemical measurements, including DPV and CV. The bare carbon paste electrode (BCPE) and modified CaO carbon paste electrode (CaO/CPE) were used as working electrodes, the saturated calomel electrode as the reference electrode, and the platinum electrode as the auxiliary electrode. The Bruker TENSOR II instrument was used for the FT-IR analysis, the PerkinElmer spectrophotometer (Lamda 35) was labored for the UV–Visible absorption spectroscopy and Rigaku Smart lab automated multipurpose X-ray diffractometer utilized for XRD analysis.

### Synthesis of CaO NPs

Calcium oxide nanoparticles were synthesized using the widespread Sol–gel method. The collected clam shells were initially cleaned then washed with hot water and deionized water repetitively and dried in an oven at 100 °C for about 24 h. Later, the dried clam shells were crumpled into adequate powder and mixed with HCl and water (1:9) in a beaker until the materialization of calcium chloride. 1 M of NaOH was deliberately added until it ensured the formation of calcium hydroxide. The formed precipitate was undisturbed for 3 h in a solution and centrifuged later for 7 min at 4000 rpm. After the centrifugation, the obtained powder was washed with deionized water and kept for drying in a vacuum oven for about 2 h at 60 °C. The obtained CaO nanoparticle was further calcinated in a muffle furnace by maintaining a temperature of 1000 °C^28^ .

### Procedure for the fabrication of CaO/CPE

For the fabrication of CaO/CPE, an augmented amount of CaO was ground with 0.24 g of graphite powder in the ratio of 70:30 (w/w) in a mortar and pestle, followed by adding 20 µL of silicone oil. Further, the mechanical grinding method was utilized for 30 min to obtain a harmonized paste. The resulting modified paste was added to the cavity of the carbon paste electrode using a spatula and pressed thoroughly to obtain a smooth surface of the electrode. The other tip of the electrode was connected to an electrochemical workstation using a copper wire with alligator clip. The same procedure was followed for all the repetitive experimentations to get CaO/CPE.

## Results and discussion

### Materials characterization

**Figure 1:**
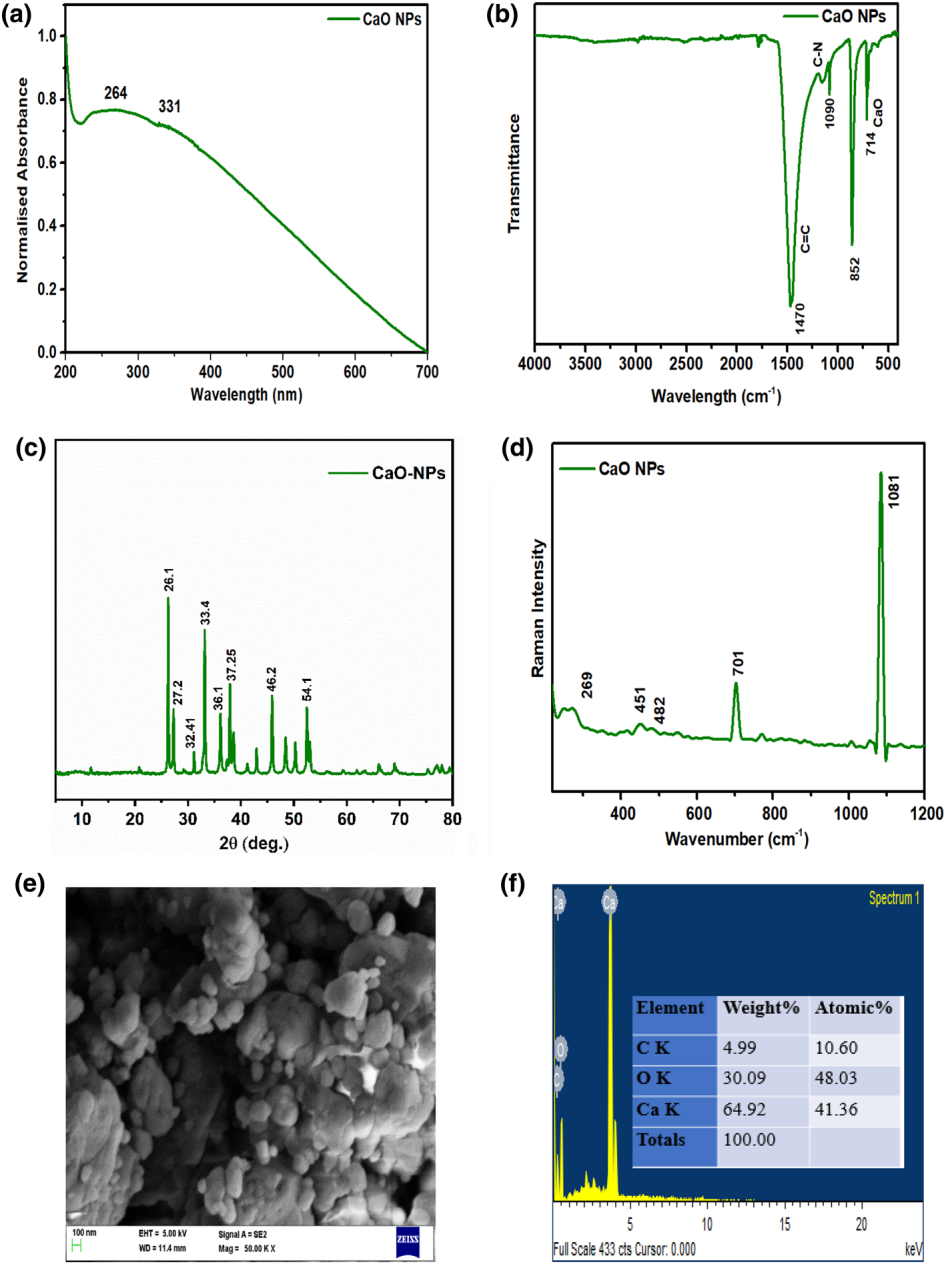
The characterization results of the CaO NPs synthesized using sol–gel route: (**a**) UV spectra, (**b**) FTIR, (**c**) XRD, (**d**) Raman, (**e**) SEM, and (**f**) EDX analysis.

The formation of Cao NPs via the co-precipitation method from the Lala clamshell was verified by UV characterization. Calcium oxide nanoparticles exhibit a shoulder peak near 331 nm and a broad-spectrum peak at 264 nm (Fig. 1a). The results are concurrent with the previous findings of Singh et al. in which biogenic materials were used as a precursor for the synthesis of CaO NPs^28^. FTIR spectrum of Cao NPs were examined to learn about the stretching and bending vibrations of the different functional groups. Figure 1b represents the FTIR spectrum of the synthesized CaO NPs. The adsorption peak at 1470 cm^-1^ corresponds to the stretching of the carbonate group present on CaO NPs. The peak at 1090 cm^-1^ is attributed to the C–N of the carboxylic group and the peak at 852 cm^-1^ corresponds to the carbonyl C=O of the nanoparticles. A sharp peak at 714 cm^-1^ is attributed to the formation of a CaO bond of the nanoparticles^28–30^. Figure 1c represents the XRD pattern of the synthesized CaO NPs. The diffraction peaks of 2θ observed at 31.035, 36.081, 51.816, 64.878 and 76.319 respectively is agreed with the recorded data for CaO (JCPDS file no. 96-900-6743). The CaO NPs good polycrystalline nature was shown by the XRD pattern's sharp peaks and shorter spectral width. However, some peaks are not indexed owning to the presence of minor impurities originated from the precursors such as minerals which are commonly present in the natural products. With a laser power and excitation source at 0.5 mW & 785 nm, the Raman spectroscopy of synthesized green CaO NPs was examined. According to Schmidt and Dariz, the dominant band in CaO at 1081 cm^-1^ is caused by calcium carbonate and it is the symmetric stretching band of the carbonate anion^31^. Calcium oxide may react with atmospheric carbon dioxide to generate calcium carbonate when heated in an environment with no pressure difference. The bonds of Ca–O play a role in the absorption at 701 cm^-1^ and 451 cm^-1^. The Raman modes of calcium carbonate can be attributed to the weak bands of about 1085 cm^-1^ and 269 cm^-1^ (Fig. 1d). Calcium oxide may be identified as the source of the resonances at 451 cm^-1^^28^. SEM was used to examine the CaO NP’ shape and texture (Fig. 1e). It showed that the calcium oxide NPs were huge agglomerates of extremely small particle materials that resembled sponges and foam. By examining the image or photograph, it can be seen that the manufactured size particles were smaller due to their surface shape. When subjected to the SEM's electron beam, the bright areas of surfaces in the image exhibit high electron emission. This demonstrated how the bright areas of surfaces had a high surface area-to-volume ratio. The manufactured particles were comprised of grains with spherical forms that had been agglomerated together, as could be seen from the microscope. The polycrystalline nature of CaO NPs is revealed by these tiny particles clumped together. Fig. 1f displays the EDX spectra of the CaO NPs. The main components of CaO NPs were calcium, carbon, and oxygen. At energy line of 5.0 keV, the characteristics of calcium, carbon, and oxygen are visible.

### Impact of CaO concentration on the oxidation behaviour of urea

**Figure 2:**
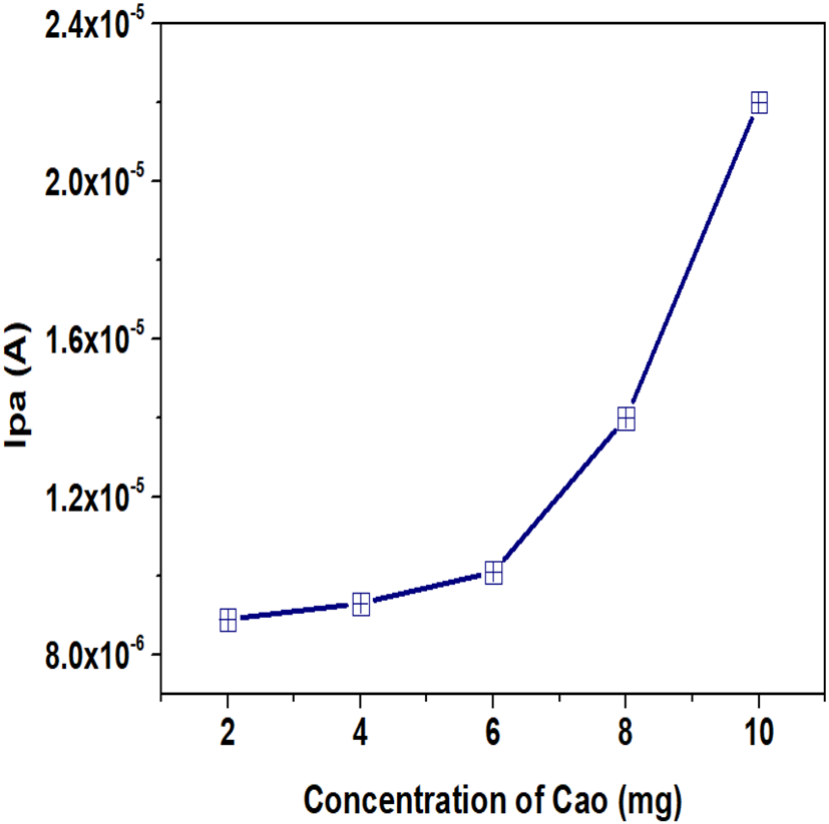
Impact of CaO concentration over the oxidation of urea.

The concentration of CaO used for the electrode modification appears to be the main factor affecting the kinetics of the electrode response. Calcium oxide nanoparticles act as a mediator for quick electron transfer between urea and the surface of the electrode assisting in rapid redox process. Hence, the working electrode was modified using different concentrations of CaO NPs (2, 4, 6, 8, and 10 mg) and employed for the measurement of urea. The current response of urea obtained due to the oxidation of urea at CaO/CPE was recorded against the modifier concentration of CaO (Fig. 2). The obtained data demonstrates that the oxidation peak current of urea increases linearly from 2 to 6 mg and further increases exponentially (with twofold enhancement compared to 6 mg) with an increase in the concentration of CaO up to 10 mg. Based on the observations made on the nature of the voltammogram, 8 mg of CaO was chosen as a modifier concentration for the development of the sensor and employed for further studies.

### Detection of Urea using CaO/CPE

**Figure 3:**
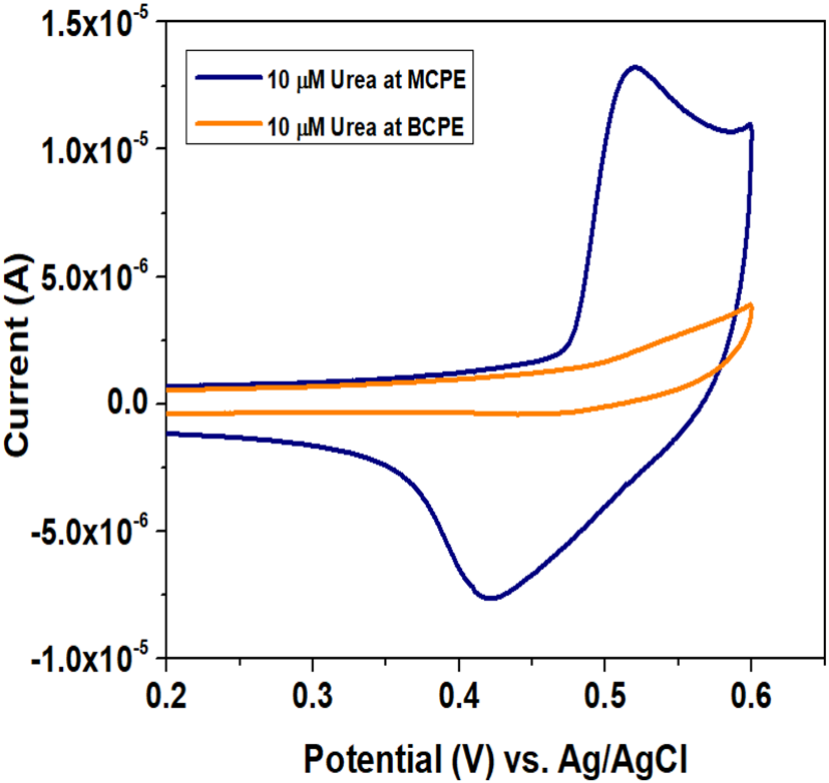
CVs measured for 10 µM urea at BCPE and CaO/CPE at scan rate 50 mV s^-1^, supporting electrolyte 0.1 M KOH in the potential range 0.2–0.6 V.

The detection procedure of urea was carried out electrochemically at the modified electrode sensor, adopting the cyclic voltammetry method. 0.1 M KOH was used as a supporting electrolyte and the scan rate was maintained at 50 mV s^-1^. Figure 3 illustrates the comparison in the detection (oxidation) of 10 µM urea at BCPE and CaO/CPE in the potential range of 0.2–0.6 V. There is no significant peak response of urea was observed in both forward and reverse scans at BCPE, may be due to the absence of active catalytic sites available on the surface of BCPE which is unable to interact with urea. After the modification with CaO NPs, an excellent redox current response of urea appeared at CaO/CPE, in which the current response was noticed to be 2.5 times higher than the BCPE.

**Scheme 1:**
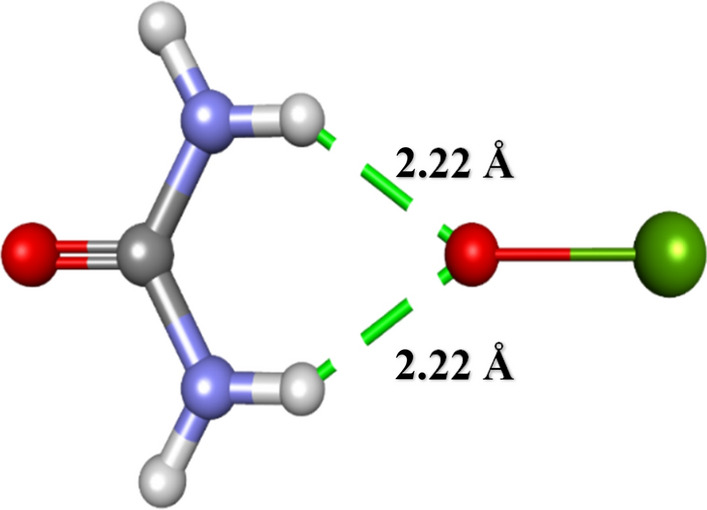
Probable interaction mechanism between CaO and urea.

The possible interactions between the urea-CaO system were predicted using the Discovery Studio visualizer (“Biovia: Discovery studio modelling environment—Google Scholar,” n.d.). Structure data files (SDF) of urea and CaO were downloaded from PubChem^33^ having CID 1176 and 14778, respectively. Then the structure of urea was loaded in the Discovery Studio visualizer, followed by applying clean geometry optimizations. The structure of CaO was also optimized similarly. Then CaO structure was added to the urea structure and again clean geometry optimizations were performed. Then CaO was selected and set as a ligand and then finally receptor-ligand interactions were displayed. The presence of two equidistant hydrogen bonds (2.22 Å) was observed between the oxygen atom of CaO and hydrogen atoms attached to the two nitrogen atoms in urea (Scheme 1). Therefore, CaO is predicted to be interacting with urea via formation of the hydrogen bonds. Hence, the electrode amended with CaO NPs enhances the electron transfer rate and acts as a sensible material for urea detection.

### Impact of sweep rate

**Figure 4:**
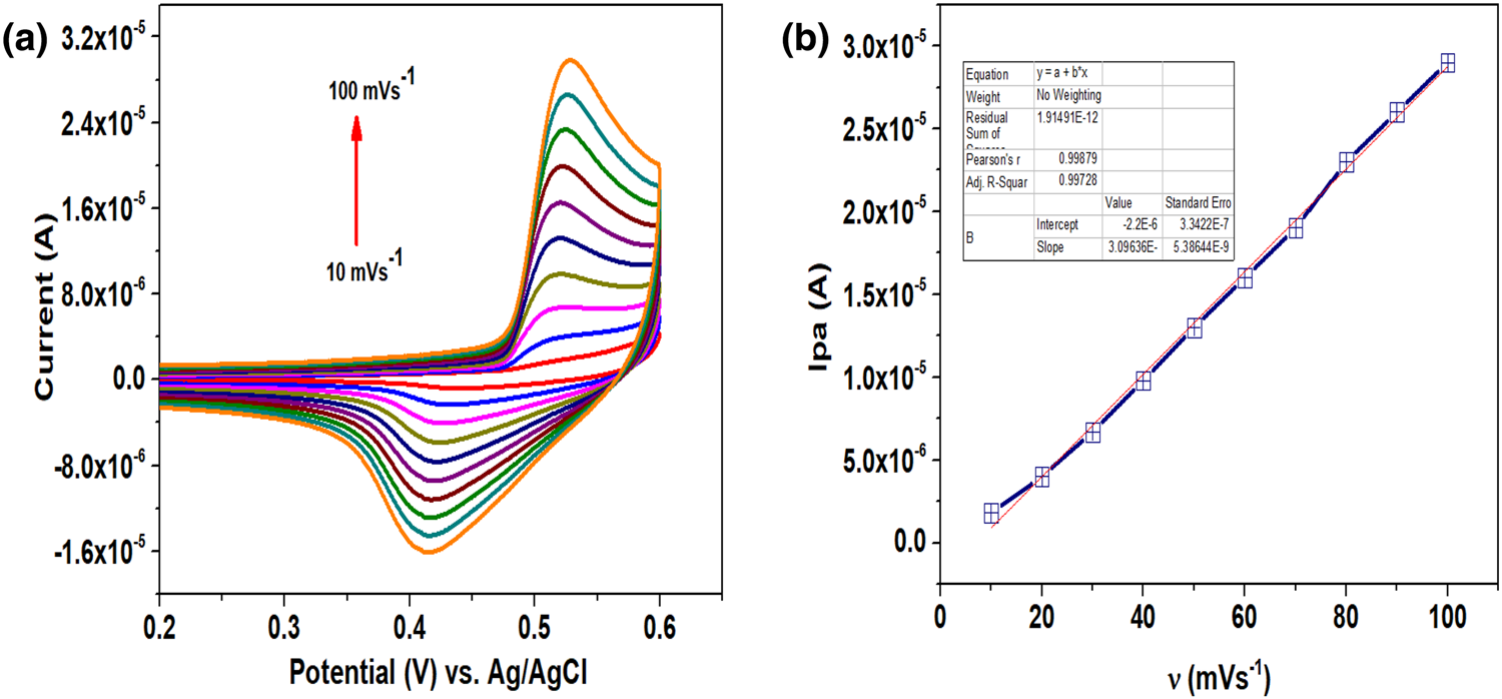
(**a**) Spectra of 10 µM urea at different scan rates ranged from 10 to 100 mV s^-1^ and (**b**) The current response (Ipa) versus sweep rate at scan rate 10–100 mV s^-1^.

By changing the sweep rate, it will be investigated if the reaction at the surface of the electrode follows the diffusion or the adsorption mechanism which is observed by the slight shift in anodic potential. This is attributed to the double layer formation or otherwise adsorption at the electrode surface respectively. The impact of sweep rate on the electrochemical determination of urea was tested at CaO/CPE using the CV technique. During the electrochemical reaction, the scan rate fluctuated from 10 to 100 mV s^-1^ and the resultant peak current response of 10 µM urea was recorded (Fig. 4a). The transfer of electrons at the electrode interface solution at the applied potential is linearly proportional to the speed of the reaction. As a result, both the oxidation and reduction peaks of urea increase with an increase in the sweep rate. Excellent linearity was observed between the current response attained versus the sweep rate (Fig. 4b). The linear regression equations for the oxidation and reduction peak currents are Ipa = 3.09E^-9^ ν–2.2E^-6^, and Ipc = 4.11E^-6^ ν–1.43E^-5^, respectively. Hence, based on the linear regression coefficients (R^2^ = 0.99), the electrode process was concluded as adsorption-controlled.

### Impact of concentration

**Figure 5:**
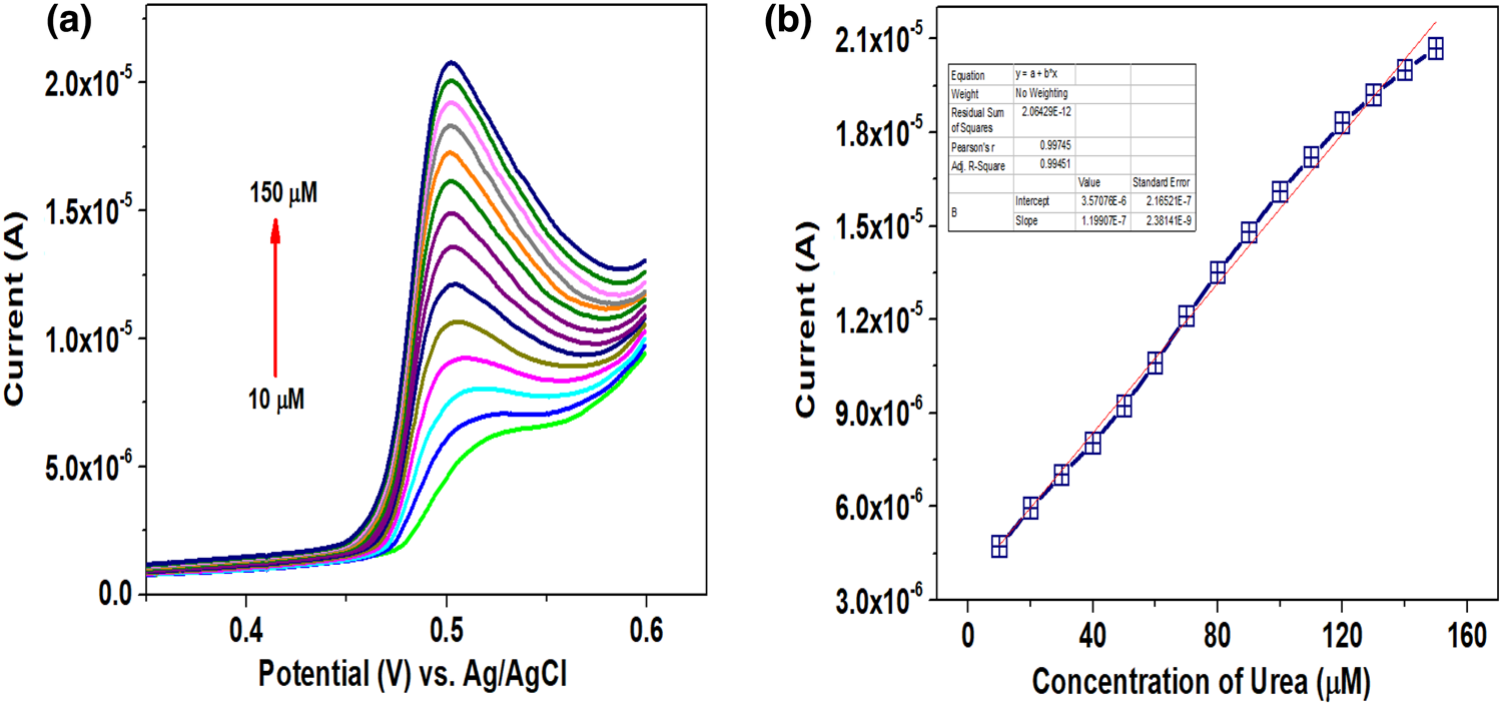
(**a**) DPVs of urea at different concentrations (10 to 150 µM) with a scan rate 50 mVs^-1^, and (**b**) Ipa versus urea concentration ranged from 10 to 150 µM with a scan rate 50 mVs^-1^.

The DPV technique was used for the quantitative analysis of urea at the modified electrode due to its exceptional sensitivity and lower detection limit capabilities than CV. The dependency of urea oxidation peak on the concentration variation from 10 to 150 µM with a scan rate 50 mVs^-1^ was examined, and the responses are shown in Fig. 5a. The magnitude of oxidative current is linearly increased with the increase in the urea concentration indicating the excellent catalytic behaviour of CaO NPs without fouling effect. Additionally, the linearity of the oxidation peaks altered as the urea concentration increased, which supports the claim that the modified electrode is efficiently electro-catalysed the urea through the diffusion-controlled mechanism. The linear correlation between the concentration and anodic peak current I_pc_ = 1.19E^-7^ C (µM) + 3.57E^-6^; R^2^ = 0.99 is illustrated in Fig. 5b. Lower detection limit of the sensor was determined^34^ using Eq. (1) and assessed to be 0.032 µM.

1 $$\mathrm{LLOD}=3\mathrm{S}/\mathrm{M}$$

where S is the standard deviation derived from the blank, and M is the slope derived from the peak current (Ip) versus concentration (C). The LLOD achieved was compared with similar works reported previously and tabulated in Table 1. Hence, CaO/CPE showed excellent behaviour as a sensor for urea detection.

**Table 1:** Comparision of CaO/CPE with previously reported articles for urea detection.

Modified electrodes	Linear range (µM)	Detection method	LOD (µM)	Real sample	References
Gr-PANi/GCE	10–200	I-V measurements	5.88	Milk, tap water	^7^
Pt nanoflower-PANi-urease/GCE	100–1000	Amperometry	10	–	^35^
PPy/Pt	80–1440	Amperometry	40	Unknown concentration of urea	^36^
PPy/Polyion complex/Pt	30–30,000	CV	20	–	^37^
Urease/PPy/CP	1.22–3.85	Amperometry	1	Serum	^38^
Ni-MOF/Nafion/GCE	10–7000	Amperometry	2.71	Urine samples	^39^
NiO/CTAB/GO/GC	100–1200	Amperometry	8	Mineral water, seawater, and tap water	^40^
ZnO NRs	1–24,000	CV	10	Urea	^41^
Urs/H40–Au/ITO	10–35,000	Photometry	10	Blood serum and urine	^42^
CaO/CPE	10–150	DPV	0.032	Tap water and fresh liquid milk	Present work

### Investigation of reproducibility, stability, and selectivity of CaO/CPE

**Figure 6:**
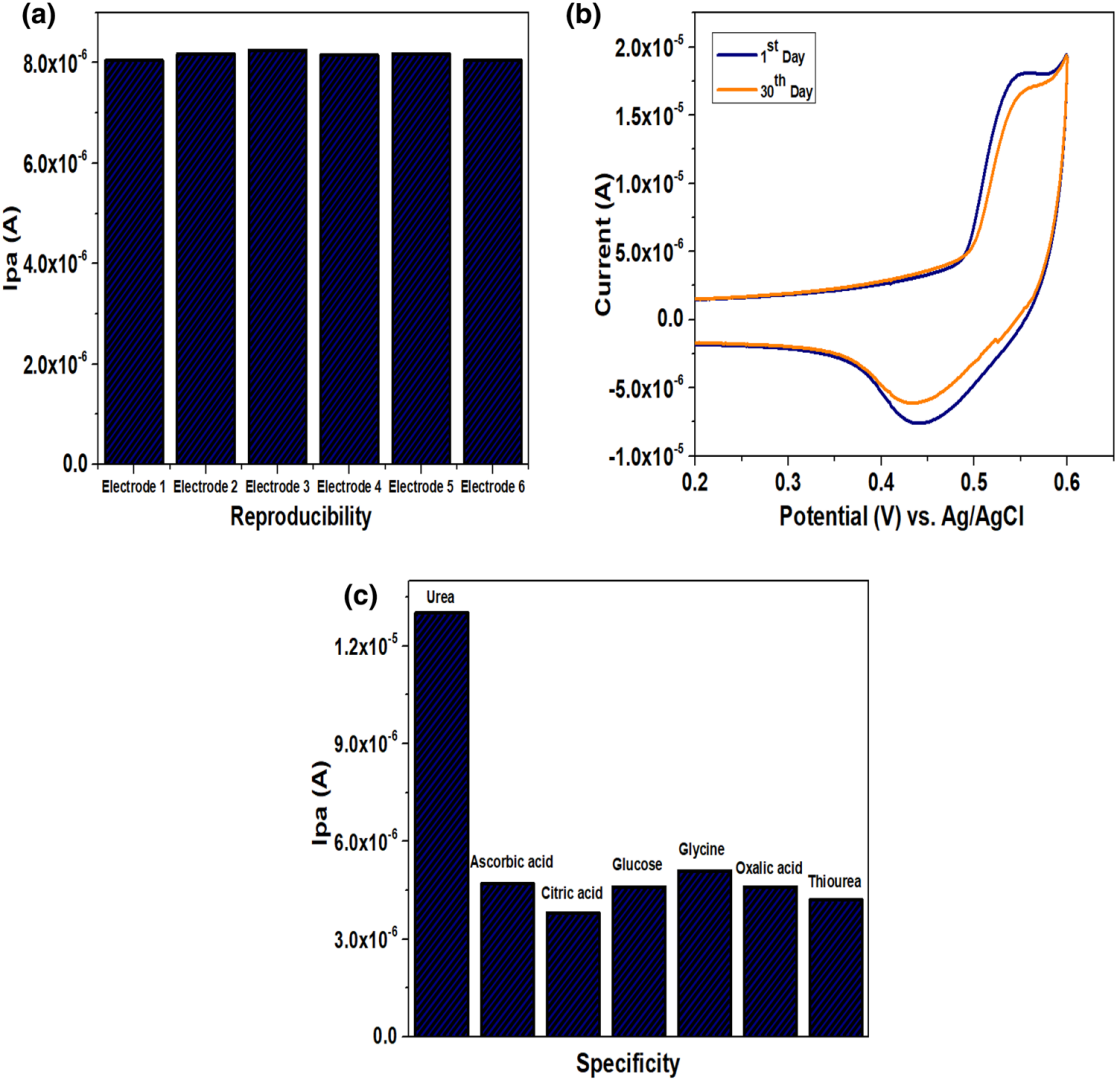
(**a**) Current response measured for 10 µM urea at six different modified electrodes, (**b**) CVs of urea measured in several days, (**c**) Selectivity of the CaO/CPE for urea detection in presence of other interferants (ascorbic acid, citric acid, glucose, glycine, oxalic acid and thiourea).

The reproducibility of the sensor was evaluated by testing the six different electrodes in urea solution (10 µM) using KOH (0.1 M) as a supporting electrolyte. The relative standard deviation of the oxidative peak current of these electrodes was around ± 3.8% (Fig. 6a), which indicates that the urea detection using different electrodes is reproducible. The stability of the modified electrode was investigated in terms of shelf life. The modified electrode was prepared and stored under a vacuum to prevent oxidation of the sensing material. The oxidative peak current of urea was recorded on the 1st and 30th day and the outcome was depicted in Fig. 6b. The results indicate that, even after 30 days, there was a negligible reduction in the peak response (RSD =  ± 3.18%) compared with the initial current intensity, suggests the excellent stability of the CaO/CPE. Since both ascorbic acid and glucose are prevalent components of human blood and are essential for metabolic activities, the selectivity of the modified electrode for urea detection in the presence of AA, and glucose along with other inferring molecules was assessed. Interferants like citric acid, glycine, oxalic acid, and thiourea of equimolar concentration (100 µM) were chosen for the analysis. As shown in Fig. 6c, the current response of urea (10 µM) is more significant compared to other interfering molecules, suggesting the negligible impact of the interferents on the determination of urea at the modified electrode. Hence, the developed sensor showed magnificent interference-free behaviour for the detection of urea.

### Real-world sample analysis

Detection of urea in tap water and milk samples was carried out using the DPV technique to investigate the sensor’s potential for usage in medical diagnostics and food safety. Urea-spiked tap water and milk samples at various concentrations were prepared. The DPV responses corresponding to urea oxidation for different concentrations of water and milk samples in 0.1 M KOH supporting electrolytes were obtained. The measured urea concentration was computed and compared to the actual spiking concentration using the calibration plot, and the recovery rates obtained were tabulated in Table 2. The results demonstrate a recovery rate between 97.8 and 99.5%, which indicates the suitability of the sensor for detecting urea in milk samples. Hence, CaO/CPE could be effectively employed for the detection of urea in real-world sample analysis.

**Table 2:** Data recorded for the detection of urea in milk samples at CaO/CPE.

Sample	Added (µM)	Found (µM)	Recovery (%)
Fresh liquid milk	10	9.92	99.2
	20	19.7	98.9
Tap water	10	9.95	99.5
	20	19.56	97.8

## Conclusions

In summary, a sensitive and green CaO NPs -based electrochemical sensor was developed using an eco-friendly synthesis to detect urea. The designed modified electrode sensor was efficaciously used for the detection of urea. A well-defined linear response range was obtained for urea oxidation with an acceptable limit of detection at CaO/MCPE. The electrode process was investigated, in which adsorption-controlled phenomena was confirmed. The selectivity of the sensor was studied by considering the major interfering molecules for urea oxidation. The possible interaction mechanism between CaO and urea was demonstrated. Also, the real-time applicability of the sensor was researched by detecting the urea in milk samples, in which tolerable recovery was achieved. Hence, the invented CaO-modified electrode sensor can be successfully used in food safety applications to monitor the urea level. Further, the proposed methodology in this work can be implemented and scaled up to develop and fabricate portable, flexible, economic and handheld sensors in order to monitor the presence of urea quantitatively and qualitatively in milk samples (Supplementary file 1).

### Supplementary Information

Supplementary Information 1.

## Data Availability

The data that support the findings of this study are available from [Praveen C. Ramamurthy]. Still, restrictions apply to the availability of these data, which were used under license for the current study, and so are not publicly available. However, data are available from the authors upon reasonable request and with permission of [Praveen C. Ramamurthy].

## References

[1] Yin W (2021). Urea detection in milk by urease-assisted pH-sensitive carbon dots. Appl. Opt..

[2] Poonia A (2017). Detection of adulteration in milk: A review. Int. J. Dairy Technol..

[3] Handford CE, Campbell K, Elliott CT (2016). Impacts of milk fraud on food safety and nutrition with special emphasis on developing countries. Compr. Rev. Food Sci. Food Saf..

[4] Kumar V, Dash S (2021). Evaporation-based low-cost method for the detection of adulterant in milk. ACS Omega.

[5] Trivedi UB (2009). Potentiometric biosensor for urea determination in milk. Sens. Actuators B Chem..

[6] Food Safety and Standards (Food Products Standards and Food Additives) Regulations, 2011. (2022).

[7] Sha R, Komori K, Badhulika S (2017). Graphene-Polyaniline composite based ultra-sensitive electrochemical sensor for non-enzymatic detection of urea. Electrochim. Acta.

[8] Lau WL, Vaziri ND (2017). Urea, a true uremic toxin: The empire strikes back. Clin. Sci..

[9] Khan KM, Krishna H, Majumder SK, Gupta PK (2015). Detection of urea adulteration in milk using near-infrared raman spectroscopy. Food Anal. Methods.

[10] Chaudhari R, Joshi A, Srivastava R (2017). PH and urea estimation in urine samples using single fluorophore and ratiometric fluorescent biosensors. Sci. Rep..

[11] MacMahon S, Begley TH, Diachenko GW, Stromgren SA (2012). A liquid chromatography–tandem mass spectrometry method for the detection of economically motivated adulteration in protein-containing foods. J. Chromatogr. A.

[12] Jha SN, Jaiswal P, Borah A, Gautam AK, Srivastava N (2015). Detection and quantification of urea in milk using attenuated total reflectance-fourier transform infrared spectroscopy. Food Bioproc. Tech..

[13] Sharma S (2021). Investigation of adulteration in milk using surface plasmon resonance. ECS J. Solid State Sci. Technol..

[14] Anil AG, Sunil Kumar Naik TS, Subramanian S, Ramamurthy PC (2021). A novel non-enzymatic urea sensor based on the nickel complex of a benzimidazoyl pyridine derivative. J. Electroanal. Chem..

[15] Wang X (2021). Conductive 2D metal-organic framework (Co, NiCo, Ni) nanosheets for enhanced non-enzymatic detection of urea. Electroanalysis.

[16] Tran TQN, Das G, Yoon HH (2017). Nickel-metal organic framework/MWCNT composite electrode for non-enzymatic urea detection. Sens. Actuators B Chem..

[17] SunilKumar Naik TS, Saravanan S, Sri Saravana KN, Pratiush U, Ramamurthy PC (2020). A non-enzymatic urea sensor based on the nickel sulfide/graphene oxide modified glassy carbon electrode. Mater. Chem. Phys..

[18] Dhinasekaran D, Soundharraj P, Jagannathan M, Rajendran AR, Rajendran S (2022). Hybrid ZnO nanostructures modified graphite electrode as an efficient urea sensor for environmental pollution monitoring. Chemosphere.

[19] Singh S (2023). Effective voltammetric tool for Nano-detection of triazine herbicide (1-Chloro-3-ethylamino-5-isopropylamino-2,4,6-triazine) by naphthalene derivative. Environ. Res..

[20] Anu Prathap MU, Kaur B, Srivastava R (2019). Electrochemical sensor platforms based on nanostructured metal oxides, and zeolite-based materials. Chem. Record.

[21] Habte L (2019). Synthesis of nano-calcium oxide from waste eggshell by sol-gel method. Sustainability.

[22] Gedda G, Pandey S, Lin YC, Wu HF (2015). Antibacterial effect of calcium oxide nano-plates fabricated from shrimp shells. Green Chem..

[23] Tongwanichniyom S, Pattamapitoon T, Sangvichien N, Phornphisutthimas S (2021). Production of calcium oxide from waste oyster shells for a value-added application of antibacteria. Copyright@ EM Int..

[24] Ijaz U, Bhatti IA, Mirza S, Ashar A (2017). Characterization and evaluation of antibacterial activity of plant mediated calcium oxide (CaO) nanoparticles by employing Mentha pipertia extract. Mater. Res. Express.

[25] Jadhav V (2022). Green synthesized calcium oxide nanoparticles (CaO NPs) using leaves aqueous extract of moringa oleifera and evaluation of their antibacterial activities. J. Nanomater..

[26] Meshkatalsadat MH, Zahedifar M, Pouramiri B (2022). Facile green synthesis of CaO NPs using the Crataegus pontica C. Koch extract for photo-degradation of MB dye. Environ. Sci. Pollut. Res..

[27] Singh S (2022). A novel CaO nanocomposite cross linked graphene oxide for Cr(VI) removal and sensing from wastewater. Chemosphere.

[28] Singh S (2022). A novel CaO nanocomposite cross linked graphene oxide for Cr(VI) removal and sensing from wastewater. Chemosphere.

[29] Eljiedi, A. A. A., Kamari, A., Sunardi & Fatimah, I. Lala clam (Orbicularia orbiculata) shell as an eco-friendly adsorbent for Cd(II), Cu(II) and Pb(II) ions. **26**, 462–475. 10.1080/25765299.2019.1674046 (2019).

[30] Jalu RG, Chamada TA, Kasirajan DR (2021). Calcium oxide nanoparticles synthesis from hen eggshells for removal of lead (Pb(II)) from aqueous solution. Environ. Chall.

[31] Schmid T, Dariz P (2015). Shedding light onto the spectra of lime: Raman and luminescence bands of CaO, Ca(OH)2 and CaCO3. J. Raman Spectrosc..

[32] Biovia: Discovery studio modeling environment - Google Scholar. https://scholar.google.co.in/scholar?hl=en&as_sdt=0%2C5&q=BIOVIA%2C+Dassault+Systèmes%2C+BIOVIA+Discovery+Studio+Visualizer%2C+Version+21.1.0.20298%2C+San+Diego%3A+Dassault+Systèmes%2C+2021&btnG=.

[33] Kim S (2021). PubChem in 2021: New data content and improved web interfaces. Nucleic Acids Res..

[34] Kumar Naik TSS (2022). Low cost, trouble-free disposable pencil graphite electrode sensor for the simultaneous detection of hydroquinone and catechol. Mater. Chem. Phys..

[35] Jia W, Su L, Lei Y (2011). Pt nanoflower/polyaniline composite nanofibers based urea biosensor. Biosens. Bioelectron..

[36] Mondal S, Sangaranarayanan MV (2013). A novel non-enzymatic sensor for urea using a polypyrrole-coated platinum electrode. Sens. Actuators B Chem..

[37] Osaka T, Komaba S, Seyama M, Tanabe K (1996). High-sensitivity urea sensor based on the composite film of electroinactive polypyrrole with polyion complex. Sens. Actuators B Chem..

[38] Syu MJ, Chang YS (2009). Ionic effect investigation of a potentiometric sensor for urea and surface morphology observation of entrapped urease/polypyrrole matrix. Biosens. Bioelectron..

[39] Bao C (2019). Ultrathin nickel-metal-organic framework nanobelt based electrochemical sensor for the determination of urea in human body fluids. RSC Adv..

[40] Parsaee Z (2018). Synthesis of novel amperometric urea-sensor using hybrid synthesized NiO-NPs/GO modified GCE in aqueous solution of cetrimonium bromide. Ultrason. Sonochem..

[41] Ahmad R, Tripathy N, Hahn YB (2014). Highly stable urea sensor based on ZnO nanorods directly grown on Ag/glass electrodes. Sens. Actuators B Chem..

[42] Tiwari A, Aryal S, Pilla S, Gong S (2009). An amperometric urea biosensor based on covalently immobilized urease on an electrode made of hyperbranched polyester functionalized gold nanoparticles. Talanta.

