# Supplementary material for: Green and sustainable synthesis of CaO nanoparticles: Its solicitation as a sensor material and electrochemical detection of urea
Source: Sci Rep. 2023 Nov 15;13:19995. doi: 10.1038/s41598-023-46728-2 (PMC10651922; doi:10.1038/s41598-023-46728-2)
Supplement: Supplementary file 1 — Supplementary Information 1. [file 41598_2023_46728_MOESM1_ESM.docx]

**Environmental Implication (maximum limit:100 words)**

In this work, CaO NPs have been synthesized utilizing a green precursor, Lala clam seashells, by sol-gel method followed by calcination. Seashell-extracted CaO NPs were then characterized by various techniques such as scanning electron microscopy (SEM), X-ray diffraction (XRD), and fourier transform-infrared spectroscopy (FTIR). Further, CPE was modified with sea shell extracted CaO-NPs for the electrochemical sensing of urea using CV and DPV techniques. The developed sensor for urea demonstrated remarkable sensitivity, with excellent reproducibility, and was tested on real-world samples as well. In addition, the conceivable interaction sites between the urea and CaO were prophesied using the Discovery Studio visualizer.
